# Genomic and Transcriptomic Investigation of the Physiological Response of the Methylotroph *Bacillus methanolicus* to 5-Aminovalerate

**DOI:** 10.3389/fmicb.2021.664598

**Published:** 2021-04-30

**Authors:** Carsten Haupka, Luciana F. Brito, Tobias Busche, Daniel Wibberg, Volker F. Wendisch

**Affiliations:** ^1^Genetics of Prokaryotes, Faculty of Biology, CeBiTec, Bielefeld University, Bielefeld, Germany; ^2^Department of Biotechnology and Food Science, Norwegian University of Science and Technology, Trondheim, Norway; ^3^Technology Platform Genomics, Center for Biotechnology, Bielefeld University, Bielefeld, Germany; ^4^Genome Research of Industrial Microorganisms, Center for Biotechnology, Bielefeld University, Bielefeld, Germany

**Keywords:** *Bacillus methanolicus*, physiology, 5-aminovalerate, bioplastics, differential RNA-Seq, whole-genome sequencing, adaptive laboratory evolution, methylotroph

## Abstract

The methylotrophic thermophile *Bacillus methanolicus* can utilize the non-food substrate methanol as its sole carbon and energy source. Metabolism of L-lysine, in particular its biosynthesis, has been studied to some detail, and methanol-based L-lysine production has been achieved. However, little is known about L-lysine degradation, which may proceed via 5-aminovalerate (5AVA), a non-proteinogenic ω-amino acid with applications in bioplastics. The physiological role of 5AVA and related compounds in the native methylotroph was unknown. Here, we showed that *B. methanolicus* exhibits low tolerance to 5AVA, but not to related short-chain (C4–C6) amino acids, diamines, and dicarboxylic acids. In order to gain insight into the physiological response of *B. methanolicus* to 5AVA, transcriptomic analyses by differential RNA-Seq in the presence and absence of 5AVA were performed. Besides genes of the general stress response, RNA levels of genes of histidine biosynthesis, and iron acquisition were increased in the presence of 5AVA, while an Rrf2 family transcriptional regulator gene showed reduced RNA levels. In order to test if mutations can overcome growth inhibition by 5AVA, adaptive laboratory evolution (ALE) was performed and two mutants—AVA6 and AVA10—with higher tolerance to 5AVA were selected. Genome sequencing revealed mutations in genes related to iron homeostasis, including the gene for an iron siderophore-binding protein. Overexpression of this mutant gene in the wild-type (WT) strain MGA3 improved 5AVA tolerance significantly at high Fe^2+^ supplementation. The combined ALE, omics, and genetics approach helped elucidate the physiological response of thermophilic *B. methanolicus* to 5AVA and will guide future strain development for 5AVA production from methanol.

## Introduction

The production of bio-based plastics is predicted to increase in the recent future^1^. Polyamides belong to plastics, and they can be synthesized chemically in two ways: (1) via anionic ring-opening polymerization of lactams derived from ω-amino acids and (2) condensation of diamines with dicarboxylic acids. Nowadays, petrochemical production of these monomeric precursors of polyamides from crude oil and natural gas as raw materials prevails. Biotechnology offers the possibility to produce identical monomeric precursors from renewable resources as drop-in chemicals.

Microorganisms used in biotechnological industry at large scale, e.g., *Escherichia coli* and *Corynebacterium glutamicum*, an industrial producer of about 2.6 million tons of L-lysine in 2018 (Wendisch, 2020), are a suitable choice for the sustainable production of polyamide precursors by fermentation. The fermentative production of C4 and C5 dicarboxylic acids, for example, has been achieved with metabolically engineered *E. coli* and *C. glutamicum* strains (Pérez-García et al., 2018; Rohles et al., 2018; Chae et al., 2020). *C. glutamicum* strains overproducing L-lysine and L-ornithine have been engineered to overproduce the diamines cadaverine and putrescine, respectively, by pathway extension using L-lysine decarboxylase and L-ornithine decarboxylase (Mimitsuka et al., 2007; Kind et al., 2011; Schneider et al., 2012; Nguyen et al., 2015). The C4 ω-amino acid γ-aminobutyrate (GABA) can be produced by *E. coli* and *C. glutamicum* strains overproducing L-glutamate and expressing a glutamate decarboxylase (Takahashi et al., 2012; Xiong et al., 2017) or by extending the putrescine production pathway by heterologous expression of putrescine transaminase and γ-aminobutyraldehyde dehydrogenase genes (Jorge et al., 2016). *C. glutamicum* strains overproducing L-lysine were engineered for production of 5-aminovalerate (5AVA), and three alternative biosynthesis pathways were established (Supplementary Figure 1; Shin et al., 2016; Pérez-García et al., 2018; Haupka et al., 2020).

The fermentation industry mostly relies on sugars as feedstock. However, it is imperative to develop large-scale fermentation processes that do not rely on substrates with competing uses in the feed and food industries. To this end, the alternative feedstock concept was followed, and a variety of microbial strains were constructed to enable the utilization of various renewable carbon sources (Wendisch et al., 2016). For example, *C. glutamicum* was engineered to utilize xylose, arabinose, glycerol, hemicellulosic and cellulosic hydrolysates, grass juice, etc. (Rittmann et al., 2008; Gopinath et al., 2011; Sasaki et al., 2011; Schneider et al., 2011; Sgobba et al., 2018). Fermentative production of 5AVA has been reported using glucose, starch, glucosamine, xylose, arabinose, and *Miscanthus* hydrolysate as feedstocks (Joo et al., 2017; Jorge et al., 2017).

Methanol has not yet been used for fermentative production of 5AVA. At a current price of 399 USD/metric tons^2^, which is expected to drop further in the future, it is an interesting feedstock. Methanol can be produced from carbon dioxide and photochemically or electrochemically synthesized hydrogen (Cotton et al., 2020); thus, it does not have competing uses as food or feed. Natural microorganisms that can grow with methanol as the sole source of carbon and energy are well known. These methylotrophs comprise yeasts, Gram-negative bacteria, and Gram-positive bacteria (Chistoserdova, 2015). The Gram-positive, thermophilic *Bacillus methanolicus* can utilize methanol as its sole carbon and energy source, supporting fast growth at its optimal temperature of 50°C (Müller et al., 2015a). The wild-type (WT) strain *B. methanolicus* MGA3 is able to overproduce 59 g/L of L-glutamate, and it has been engineered for methanol-based production of 65 g/L of L-lysine (Brautaset et al., 2010). Introduction of genes coding for L-glutamate decarboxylase and L-lysine decarboxylase converted this strain to produce 11.3 g/L of cadaverine and 9 g/L of GABA, respectively, in fed-batch fermentations from methanol (Nærdal et al., 2015; Irla et al., 2017). Methanol-based production of acetoin was achieved by heterologous expression of genes for two decarboxylating enzymes, acetolactate synthase, and acetolactate decarboxylase (Drejer et al., 2020). Biochemical, genetic, and omics analyses provided a sound basis for the biochemical and genetic understanding of *B. methanolicus* (Müller et al., 2015a; Delépine et al., 2020), and metabolic fluxes during growth with methanol, mannitol, and arabitol have been unraveled (Müller et al., 2015b; Delépine et al., 2020). However, an insight into the response of this bacterium to non-native chemical compounds, such as cadaverine, GABA, and acetoin that were synthesized upon introduction of non-native enzymes, typically does not exist. Since *B. methanolicus* is used for the methanol-based production of proteins (Irla et al., 2020), amino acids, and derived chemicals, the response of *B. methanolicus* to the non-native L-lysine-derived ω-amino acid 5AVA has been studied.

## Materials and Methods

### Microorganisms and Cultivation Conditions

For cloning, the *E. coli* DH5α strain was exploited as a host (Hanahan, 1985) and grown in lysogeny broth (LB) at 37°C, which was supplemented with 100 μg/ml of ampicillin when required. *B. methanolicus* MGA3 strains were cultivated at 50°C in MVcM minimal medium (Brautaset et al., 2003) supplemented with 0.25 g/L of yeast extract (MVcMY), 25 μg/ml of kanamycin, and 0.5% (w/v) xylose when appropriate. All bacterial strains and plasmids are listed in Table 1. For growth experiments with *B. methanolicus*, overnight cultures in 10 ml of MVcMY were harvested and washed in the MVcMY medium before inoculation to an OD_600_ of 0.05 and supplementation with 200 mM methanol as a carbon source. Cells were cultivated in 10 ml Duetz microtiter plates (MTPs, Kuhner AG, Birsfelden, Schweiz) with culture volumes of 3 ml at 200 rpm in an Ecotron ET25-TA-RC (INFORS HT, Einsbach, Germany). Growth was monitored by determination of the OD_600_ with a V-1200 spectrophotometer (VWR, Radnor, PA, United States).

**TABLE 1:** Strains and plasmids used in this study.

Strain or plasmid	Relevant characteristics	References
**Strains**		
*E. coli* DH5α	Δ*lac*U169 (φ80*lac*Z ΔM15), *sup*E44, *hsd*R17, *rec*A1, *end*A1, *gyr*A96, *thi*-1, *rel*A1	Hanahan, 1985
*B. methanolicus* WT	WT strain MGA3, ATCC 53907	Schendel et al., 1990
AVA6	WT isolate after six ALE passages	This study
AVA10	WT isolate after 10 ALE passages	This study
XP03040	WT transformed with pBV2xp-03040^T132I^	This study
XP13980	WT transformed with pBV2xp-13980^*150E^	This study
XP14080	WT transformed with pBV2xp-14080^H116N^	This study
**Plasmids**		
pBV2xp	Amp^R^, Kan^R^, *B. methanolicus*/*E. coli* shuttle vector, pHCMC04 derivative, P_xyl_ from *B. megaterium*	Drejer et al., 2020
pBV2xp-03040^T132I^	pBV2xp for xylose inducible expression of BMMGA3_RS03040 from *B. methanolicus* AVA10	This study
pBV2xp-FepB^*150E^	pBV2xp for xylose inducible expression of BMMGA3_RS13980 from *B. methanolicus* AVA10	This study
pBV2xp-IscR^H116N^	pBV2xp for xylose inducible expression of BMMGA3_RS14080 from *B. methanolicus* AVA10	This study

### Recombinant DNA Work

Isolation of genomic DNA of *B. methanolicus* was performed by using the NucleoSpin Microbial DNA Kit (Macherey-Nagel, Düren, Germany). Classical methods, which include plasmid isolation, molecular cloning, and heat-shock transformation of *E. coli*, were performed as described previously (López et al., 2019). ALLin HiFi DNA Polymerase (highQu, Kraichtal, Germany) was used to amplify DNA sequences with genomic DNA as template. To overexpress mutated versions of genes BMMGA3_RS03040, BMMGA3_RS13980, and BMMGA3_RS14080, the respective genes were amplified from genomic DNA of *B. methanolicus* adaptive laboratory evolution (ALE) mutant AVA10, using the respective primers (Table 2). Amplified DNA fragments were joined into *Bam*HI-linearized pBV2xp through isothermal DNA assembly (Gibson et al., 2009). All cloned DNA fragments were verified by sequencing in the CeBiTec sequencing facility. *B. methanolicus* WT was transformed with the constructed plasmids and pBV2xp as described previously (Jakobsen et al., 2006).

**TABLE 2:** Primer sequences used in this study.

Primer	Sequence [5′ 3′]	Characteristics
03040_F	tgatggataaacttgttcacaaggaggtagtacatatgtcaacgaagaacaaagacaaaattattgaaagcgttcc	Amplification of BMMGA3_RS03040 for pBV2xp-03040^T132I^ (fw), sequencing
03040_R	gtacggatcccatttcccccttaagttaatggataccattcatataacgtttcataatcgg	Amplification of BMMGA3_RS03040 for pBV2xp-03040^T132I^ (rv), sequencing
03040S_F	attatcattggtatcggacttttaaagccaaatg	Amplification of BMMGA3_RS03040 SNP for pBV2xp-03040^T132I^ (fw), sequencing
03040S_R	gtccgataccaatgataattaagccc	Amplification of BMMGA3_RS03040 SNP for pBV2xp-03040^T132I^ (rv), sequencing
13980_F	tgatggataaacttgttcacaaggaggtagtacatttgaaaaaattaaaatcgctatttgctattatttctttattcacg	Amplification of BMMGA3_RS13980 for pBV2xp-FepB^*150E^ (fw), sequencing
13980_R	gtacggatcccatttcccccttaagttaatttgtgagcattattttttcaagttcatctagc	Amplification of BMMGA3_RS13980 for pBV2xp-FepB^*150E^ (rv), sequencing
13980S_F	ctgttttttcagaaactctcagaggagattgg	Amplification of BMMGA3_RS13980 SNP for pBV2xp-FepB^*150E^ (fw), sequencing
13980S_R	gagagtttctgaaaaaacagttggagcaatcg	Amplification of BMMGA3_RS13980 SNP for pBV2xp-FepB^*150E^ (rv), sequencing
14080_F	tgatggataaacttgttcacaaggaggtagtacatttgaatagtgatttttctattgctgtccattgcg	Amplification of BMMGA3_RS14080 for pBV2xp-IscR^H116N^ (fw), sequencing
14080_R	gtacggatcccatttcccccttaagctcattgacaacctccttaactgaaac	Amplification of BMMGA3_RS14080 for pBV2xp-IscR^H116N^ (rv), sequencing
14080S_F	catttttgaagaagctgaggaaaatc	Amplification of BMMGA3_RS14080 SNP for pBV2xp-IscR^H116N^ (fw), sequencing
14080S_R	cgtaatccgttttaaataattcatcagattttcctc	Amplification of BMMGA3_RS14080 SNP for pBV2xp-IscR^H116N^ (rv), sequencing
PRI_F	tgatggataaacttgttcacaaggaggtagtacatatgaagaagcttacttttgtcggagc	Amplification of *proI* after RNA isolation (fw)
PRI_R	gtacggatcccatttcccccttaagttactgctttactgttactgcttctgtg	Amplification of *proI* after RNA isolation (rv)
SPA_F	tgcatgcctgcaggtcgactgcagggatcgggacaatgac	Amplification of DNA upstream of *spo0A* after RNA isolation (fw)
SPA_R	cccatccactaaacttaaacacacaccgactacttccatatcatcc	Amplification of DNA upstream of *spo0A* after RNA isolation (rv)

*fw, forward primer; rv, reverse primer.*

### Transcriptomics: Cultivation and RNA Isolation

*Bacillus methanolicus* cultures were grown in MVcM or MVcMY media containing 200 mM methanol supplemented with or without 50 mM 5AVA, respectively. Cells were harvested in the mid-log phase at an OD_600_ of 0.6, and isolation of total RNA was performed individually for each cultivation condition as described previously (López et al., 2019). The RNA samples were tested for contamination with DNA using primers PRI_F and PRI_R for the amplification of the *proI* gene and primers SPA_F and SPA_R for the amplification of the *spo0A* gene (Table 2). No product was obtained for any of the tested RNA samples (data not shown). Further quality control was conducted as described previously (López et al., 2019) before processing of the RNA samples for differential RNA-Seq analysis.

### Transcriptomics: Preparation of cDNA and Differential RNA-Seq

Isolated RNA samples from *B. methanolicus* MGA3 were used in biological triplicates for the cDNA library preparation. A Ribo-Zero rRNA removal kit (bacteria) from Illumina (San Diego, CA, United States) was used to remove the ribosomal RNA molecules from the isolated total RNA. Removal of rRNA was checked by an Agilent RNA Pico 6000 kit on an Agilent 2100 Bioanalyzer (Agilent Technologies, Böblingen, Germany). RNA was free of detectable rRNA. Preparation of cDNA libraries was performed according to the manufacturer’s instructions for the TruSeq stranded mRNA kit (Illumina, San Diego, CA, United States). Subsequently, each cDNA library was sequenced on a HiSeq 1500 (2 × 75 nt PE rapid v2) sequencer system (Illumina, San Diego, CA, United States). RNA-Seq raw data files are available in the ArrayExpress database under accession number E-MTAB-10101. The resulting sequence reads were trimmed with Trimmomatic v0.33 (Bolger et al., 2014) to a minimal length of 35 base pairs and subsequently mapped onto the *B. methanolicus* MGA3 reference sequences of the chromosome (NZ_CP007739) and the native plasmids pBM19 and pBM69 (NZ_CP007741 and NZ_CP007740, respectively) using Bowtie 2 (Langmead and Salzberg, 2012). The ReadXplorer software version 2.0 (Hilker et al., 2016) and the integrated DESeq2 algorithm (Love et al., 2014) were used for the visualization of the mapped reads and the differential gene expression analysis, respectively. Differentially expressed targets were filtered with a baseMean ≥ 30, a log_2_ fold change ≥ | 1|, and an adjusted *P*-value ≤ 0.01. Manual sequence analysis with BLASTx (Altschul et al., 1990) was conducted for the remaining genes which were coding for hypothetical proteins.

### ALE

*Bacillus methanolicus* WT was subjected to an ALE experiment with progressively increasing 5AVA concentrations (50–400 mM) and varying passage intervals (8–72 h). The cells were grown in Duetz MTPs with a culture volume of 3 ml MVcMY supplemented with 200 mM methanol and washed in the MVcMY medium before reinoculation to an OD_600_ of 0.05. For all passages, a control without 5AVA was grown in parallel. In total, 50 μl of every second passage was plated on an SOB agar and incubated overnight at 50°C. Single colonies were reinoculated in liquid in the SOB medium. The overnight cultures were plated, and single colonies were picked, cultivated, and stored as glycerol stocks at −80°C for whole-genome sequencing. Evolved WT strains obtained after six passages (AVA6) and ten passages (AVA10) were selected for further investigation.

### Multi-Tolerance Analysis

The tolerance to several analytes was assessed for *B. methanolicus* WT and ALE strain AVA10 by monitoring the growth of the respective strains in Duetz MTPs with a culture volume of 3 ml MVcMY supplemented with 200 mM methanol and up to 100 mM of the analyte of interest. These included metabolites of 5AVA biosynthetic pathways, L-lysine, cadaverine, 5AVA, and glutaric acid (Pérez-García et al., 2018; Haupka et al., 2020), and structural analogs and other bioplastic precursors, GABA, 6-amino caproate (6ACA), succinate (Chae et al., 2020), and the naturally secreted product glutamate (Brautaset et al., 2003).

### Whole-Genome Sequencing

Isolated genomic DNA from *B. methanolicus* WT and ALE strains was used for whole-genome sequencing. The raw read data are available via NCBI BioProject ID PRJEB427809. DNA library preparation, trimming and mapping of the reads, and visualization were performed as described previously (Hennig et al., 2020). ReadXplorer 2.0 was used for visualization of the processed reads and detection of single-nucleotide polymorphism (SNP) in all CDSs of *B. methanolicus*. Minimal scores for base quality, average base quality, and average mapping quality were set to 20, and the minimum percentage of variation was 90, while the cutoff for the minimum number of varying bases was seven.

### Analysis of Proteins With Non-synonymous Amino Acid Exchanges

A subset of three SNPs derived from *B. methanolicus* AVA10 was computationally investigated at the protein level. The software tools Phyre2 in tandem with the in-house Missense3D and COACH-D (Kelley et al., 2015; Wu et al., 2018) were exploited for homology modeling, mutation-induced instability prediction, and ligand prediction, respectively. Genes harboring the SNPs were overexpressed from the vector pBV2xp in the WT strain. The engineered strains were cultivated in the Duetz system with a culture volume of 3 ml MVcMY supplemented with 200 mM methanol and induced with 0.5% xylose. Iron(II) sulfate and copper(II) sulfate were titrated from zero to five times of its original concentration in the MVcM recipe (Brautaset et al., 2003), respectively.

### High-Performance Liquid Chromatography (HPLC)

In order to quantify 5AVA in the cultivation medium, an HPLC system (1200 series, Agilent Technologies Deutschland GmbH, Böblingen, Germany) was used as described previously (Jorge et al., 2017). In total, 500 μl cell cultures were centrifuged at 14,000 rpm for 10 min, and the supernatant was stored at -20°C prior to analysis. After derivatization of the samples with OPA (*ortho*-phthaldialdehyde), a fluorescence detector (FLD G1321A, 1200 series, Agilent Technologies) was exploited for detection of 5AVA.

## Results

### 5AVA Impaired Growth at Low Concentrations

The biosynthesis ofL-lysine in *B. methanolicus* is known to some detail; however, L-lysine degradation has not yet been studied (Foerster, 1971; Barker et al., 1987; Revelles et al., 2004; Neshich et al., 2013). L-Lysine degradation proceeds via cadaverine and/or 5AVA to glutarate and succinate. To further our physiological understanding of L-lysine metabolism in *B. methanolicus* and as a basis for application to the methanol-based production of L-lysine and L-lysine-derived compounds, we studied the response of this methylotroph to these compounds.

**FIGURE 1:**
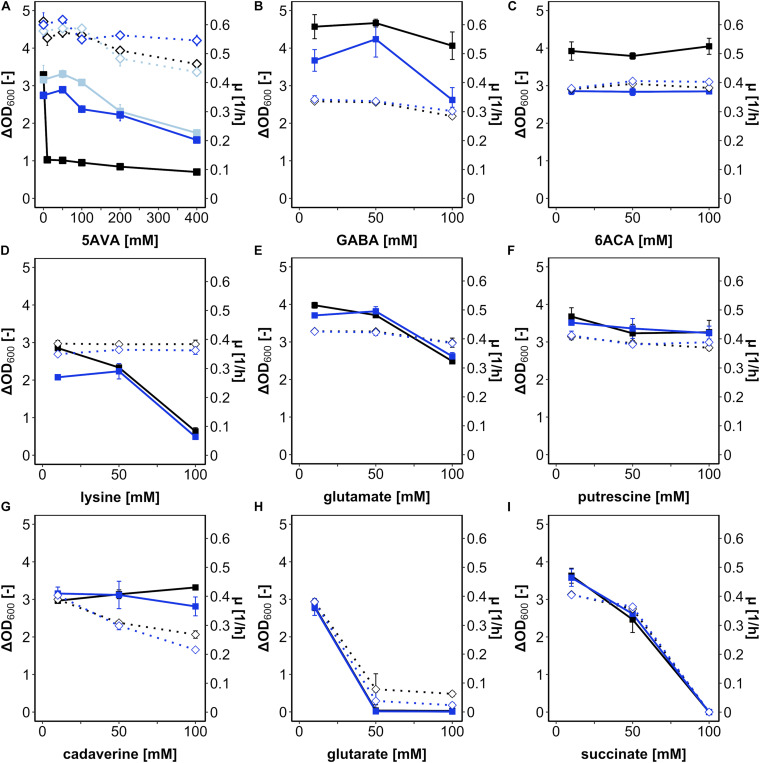
Growth of *B. methanolicus* in the presence of 5AVA **(A)**, GABA **(B)**, 6ACA **(C)**, L-lysine **(D)**, L-glutamate **(E)**, putrescine **(F)**, cadaverine **(G)**, glutarate **(H)**, and succinate **(I)**. *B. methanolicus* WT (black), AVA6 (light blue; **A**), and AVA10 (blue) were cultivated in MVcMY medium supplemented with 0, 10, 50, 100, 200, and 400 mM 5AVA **(A)** and 10, 50, and 100 mM **(B–H)** of the other compounds, respectively. The growth rates (empty diamonds, dotted lines) and ΔOD_600_ (full squares, straight lines) are shown as means and standard deviations of triplicate cultivations.

First, *B. methanolicus* was grown in a medium with methanol as a carbon and energy source in the presence of various concentrations of L-lysine and its potential degradation products (cadaverine, 5AVA, glutarate, and succinate) (Supplementary Figure 1) as well as some structural analogs differing in carbon chain lengths (the proteinogenic amino acid L-glutamate, the ω-amino acids GABA and 6ACA, and the diamine putrescine) (Figure 1). Growth was mostly impaired by 5AVA, L-lysine, glutarate, and succinate. Addition of glutarate at concentrations of 50 and 100 mM abolished growth (Figure 1H), as did succinate at 100 mM (Figure 1I). While addition of 100 mM lysine (Figure 1D) reduced the maximal biomass concentration (ΔOD_600_ value 0.6 ± 0.1), the growth rate was much less affected (Figure 1D). Notably, the only substance severely hampering growth at 10 mM was 5AVA. Although the growth rate was hardly affected, *B. methanolicus* grew poorly (ΔOD_600_ of 1.0 ± 0.1) in the presence of 10 mM 5AVA (Figure 1A). In the concentration range from 0 to 400 mM 5AVA, ΔOD_600_ was reduced from 3.2 ± 0.1 at 0 mM to 0.7 ± 0.0 at 400 mM 5AVA, whereas the growth rates were marginally reduced from 0.61 ± 0.00 h^–1^ at 0 mM 5AVA to 0.46 ± 0.00 h^–1^ at 400 mM (Figure 1A). Thus, of the tested compounds added extracellularly, 5AVA affected growth of *B. methanolicus* at the lowest concentration (10 mM). Therefore, we chose 5AVA for further studies.

**FIGURE 2:**
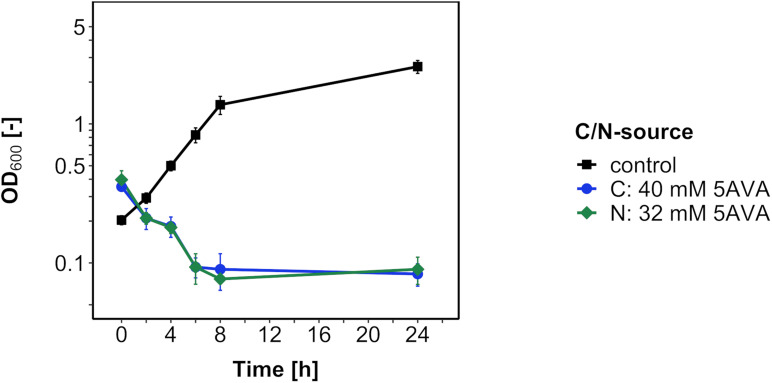
Growth of *B. methanolicus* WT with 5AVA as a carbon/nitrogen source. *B. methanolicus* WT was cultivated in MVcM medium supplemented with 5AVA as a carbon source (blue circles) and as a nitrogen source (green diamonds) or with methanol as a carbon source and ammonium sulfate as a nitrogen source (black squares). Values and error bars represent means and standard deviations of triplicate cultivations.

Next, in order to test if *B. methanolicus* catabolizes 5AVA as a carbon or nitrogen source, the medium carbon and nitrogen sources 200 mM methanol and 16 mM ammonium sulfate were replaced with 5AVA at an equimolar concentration of carbon and nitrogen, respectively (Figure 2). Because no growth was observed after 24 h, 5AVA did not support growth of *B. methanolicus* either as a sole carbon source or as a sole nitrogen source.

### Differential RNA-Seq Analysis Revealed Transcriptomic Response to 5AVA

**FIGURE 3:**
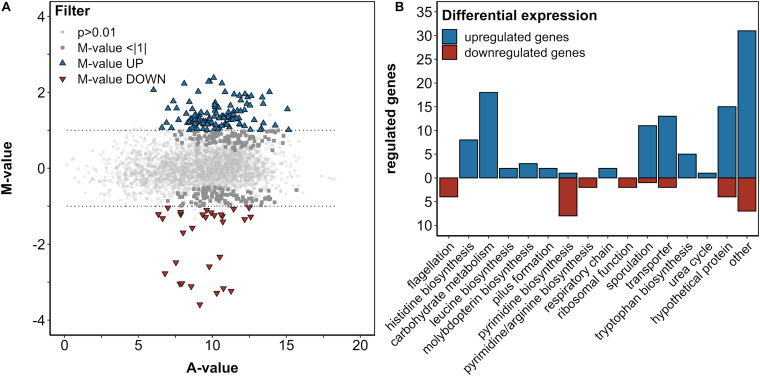
Differential gene expression analysis of *B. methanolicus* WT in the presence and absence of 50 mM 5AVA **(A)**. The log_2_-transformed M/A plot reveals upregulated (UP; blue triangles) and downregulated (DOWN; red triangles) genes with an *M*-value > | 1|, which are categorized by function **(B)**. **(A)** Differential expression is below this threshold and non-differential expressions are indicated by dark gray squares and light gray circles, respectively. Cells were cultivated in MVcMY medium to the mid-exponential growth phase, and RNA was subsequently isolated, reverse transcribed to cDNA, and sequenced in biological triplicates. Differential expression was determined by DESeq2 with an adjusted *p*-value ≤ 0.01 according to the Wald test (Love et al., 2014).

In order to investigate the transcriptomic response of *B. methanolicus* to 5AVA, a differential RNA-Seq was performed using *B. methanolicus* cells exponentially growing in the absence or presence of 50 mM 5AVA. After sequencing of enriched mRNAs, 31.2 million reads were generated, and 30.9 million processed reads could be mapped to the chromosome and both native plasmids of *B. methanolicus* MGA3. DESeq2 analysis revealed 112 significantly upregulated genes and 30 significantly downregulated genes (fold changes ≥ 2 and ≤ −2, respectively, with an adjusted *P*-value ≤ 0.01; Figure 3A and Supplementary Table 1). After BLASTx analysis, these genes were categorized based on the KEGG PATHWAY nomenclature (Kanehisa and Goto, 2000) (Figure 3B). Prominently, genes of the histidine biosynthesis and carbohydrate metabolism were upregulated. Genes concerning flagellation, pyrimidine biosynthesis, and ribosomal function were downregulated, while sporulation was upregulated. Since these genes are associated with a general stress response rather than a 5AVA-specific adaptation, they were not considered for further analysis here.

Among the genes in the category “other,” *locus* BMMGA3_RS13980 along with BMMGA3_RS13970—coding for a FepB-type iron hydroxamate siderophore (COG0614) with an in-frame stop codon and iron ABC transporter permease, respectively—was upregulated (*M* = 1.56/1.15). Siderophores are high-affinity iron-chelating compounds transporting iron across the cell membrane (Neilands, 1995; Khan et al., 2018). The respective homolog of BMMGA3_RS13980 in *Bacillus subtilis* has been described by Schneider and Hantke (1993). Therefore, we tentatively named the *B. methanolicus* protein FepB (Supplementary Table 2). On the other hand, BMMGA3_RS14080 was downregulated (*M* = -1.04). It codes for an IscR family transcriptional regulator (COG1959) of the Rrf2 superfamily. The Rrf2 family belongs to the winged helix–turn–helix superfamily of the prokaryotic transcriptional regulators that are typically affected by small-molecule ligands (Aravind et al., 2005; Shepard et al., 2011). As the homologous IscR from *E. coli* has been a dual regulator of *E. coli* FeS cluster assembly (Rajagopalan et al., 2013), we tentatively named the *B. methanolicus* protein IscR (Supplementary Table 2). Instead of verifying selected RNA-Seq results by qRT-PCR, we aimed to identify mutations improving tolerance to 5AVA in an ALE experiment and determine if an overlap between differentially expressed genes and mutations in ALE mutants exists.

### ALE Overcame 5AVA Toxicity

**FIGURE 4:**
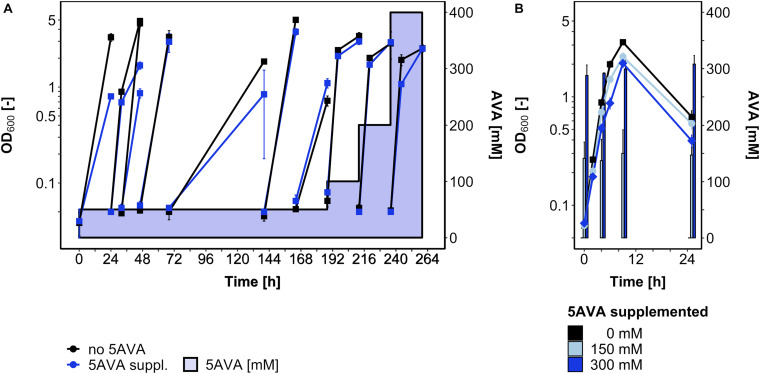
ALE for enhanced 5AVA tolerance **(A)** and growth of strain AVA10 with different 5AVA concentrations **(B)**. *B. methanolicus* cells were cultivated in MVcMY medium supplemented with various concentrations of 5AVA. **(A)** OD_600_ values during the ALE experiment with *B. methanolicus* WT are plotted for growth without (black squares) or with 5AVA (blue squares; 5AVA concentrations ranging from 50 to 400 mM indicated as blue-shaded area). Passage intervals varied (8–72 h), and every second passage was harvested and stored at -80°C after plating and isolation of a single colony. **(B)** ALE strain AVA10 was cultivated in MVcMY medium supplemented with 0 mM (black squares), 150 mM (light blue circles), and 300 mM (blue diamonds) 5AVA. The 5AVA concentration in the supernatant was determined by HPLC. Values and error bars represent means and standard deviations of triplicate cultivations.

Since some, albeit little growth (ΔOD_600_ around 1) was observed in the presence of 5AVA and the global gene expression analysis revealed induction of the general stress response upon 5AVA addition, an ALE experiment was conducted to select *B. methanolicus* mutants that withstand higher 5AVA concentrations (Figure 4A). In a serial dilution experiment, *B. methanolicus* WT was cultivated with gradually increasing 5AVA concentrations (50–400 mM). Passage intervals were altered (8–72 h) in order to allow transfers during various growth phases. In passage 6 (accordingly, a single colony isolated after plating on SOB was named strain AVA6), a maximum OD_600_ of 3.7 ± 0.3 was reached after 164 h in the presence of 50 mM 5AVA. In the 10th passage, AVA10 reached an OD_600_ of 2.5 ± 0.1 after 260 h in the presence of 400 mM 5AVA (Figure 4A).

Glycerol stocks of strains AVA6 and AVA10 were precultured on MVcMY with 200 mM methanol before being tested for growth with up to 400 mM 5AVA in comparison to *B*. *methanolicus* WT. Both ALE strains grew, e.g., to about twofold higher maximal OD_600_ than WT when supplemented with 400 mM 5AVA (Figure 1A). In addition, in the presence of 400 mM 5AVA, the growth rate of ALE strain AVA10 was increased by 20% (0.55 ± 0.02 vs. 0.46 ± 0.00 h^–1^; Figure 1A). Next, it was tested whether ALE strain AVA10 grew better when 5AVA was added to the growth medium because this strain converted or catabolized 5AVA. Strain AVA10 was cultivated in the presence of 0, 150, and 300 mM 5AVA with growth and the 5AVA concentration in the culture medium monitored over time (Figure 4B). The 5AVA concentration in the growth medium remained stable throughout the cultivation, and within 9 h, ALE strain AVA10 grew with growth rates of 0.40 and 0.38 h^–1^ to maximal OD_600_ of 2.3 and 2.0 with 150 and 300 mM 5AVA, respectively (Figure 4B). Thus, ALE allowed isolating a mutant strain with enhanced tolerance toward 5AVA that did not take up or catabolize this ω-amino acid.

### ALE Strain AVA10 Showed Tolerance Specific for 5AVA, Not for Related Compounds

*Bacillus methanolicus* ALE strain AVA10 grew well in the presence of the ω-amino acid 5AVA; however, it remained to be shown whether this tolerance was specific for 5AVA or affected growth in the presence of related compounds. To this end, growth of AVA10 in the presence of GABA (Figure 1B), 6ACA (Figure 1C), lysine (Figure 1D), glutamate (Figure 1E), putrescine (Figure 1F), cadaverine (Figure 1G), glutarate (Figure 1H), and succinate (Figure 1I) was compared to that of *B. methanolicus* WT. Growth rates and maximal OD_600_ varied in these growth experiments; however, both strains did not differ much from each other in these growth experiments when diamines, dicarboxylates of proteinogenic amino acids, were added (Figure 1). Notably, ALE strain AVA10 grew to lower maximal OD_600_ compared with *B. methanolicus* WT, when the ω-amino acids GABA (Figure 1B) and 6ACA (Figure 1C) were present, while the opposite was the case in the presence of the ω-amino acid 5AVA (Figure 1A). Thus, the ALE experiment allowed selecting a mutant that specifically evolved tolerance toward 5AVA.

### Whole-Genome Sequencing of Mutants and Overexpression Analysis

**FIGURE 5:**
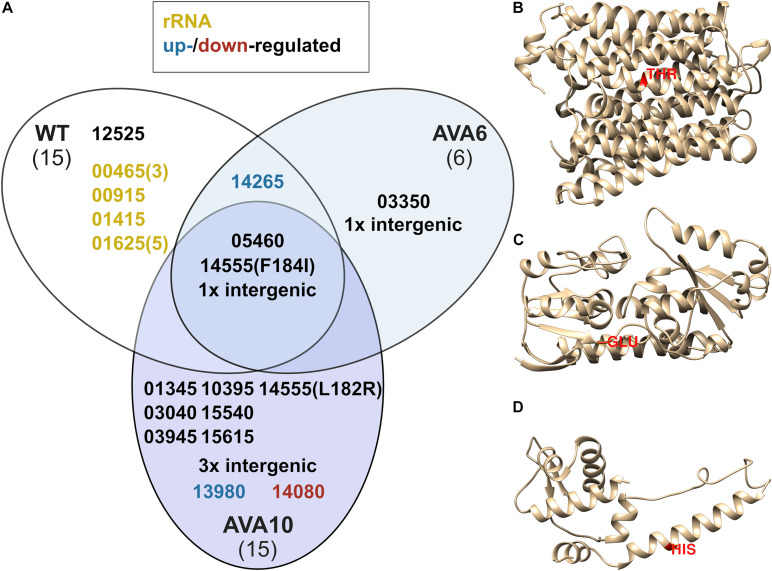
SNPs determined by whole-genome sequencing of *B. methanolicus* WT, AVA6, and AVA10 **(A)** and predicted protein structures of three key target genes BMMGA3_RS03040 **(B)**, BMMGA3_RS13980 **(C)**, and BMMGA3_RS14080 **(D)** comprising respective SNPs in AVA10. Isolated genomic DNA of *B. methanolicus* strains WT, AVA6, and AVA10 were sequenced, and SNPs in all CDSs of *B. methanolicus* were detected. **(A)** Venn diagram for observed SNPs. Gene *loci* were abbreviated such that the leading “BMMGA3_RS” was cut, leaving a five-digit identifier. Optional tailing numbers in brackets indicate multiple SNPs. Yellow identifiers refer to SNPs in genes coding for ribosomal RNA, and blue and red identifiers highlight SNPs in upregulated and downregulated genes, respectively, according to the differential RNA-Seq analysis. **(B–D)** 3D protein models were predicted using Phyre2 (Kelley et al., 2015). Selected SNPs resulting in amino acid exchanges are highlighted in red. Intergenic mutations found in AVA6 and AVA10 are described in Table 4.

In order to determine the genetic background for 5AVA tolerance, the genomes of ALE strains AVA6 and AVA10 were sequenced and compared to those of *B. methanolicus* WT. For better comparison, the genome sequence of the *B. methanolicus* WT inoculum used to start the ALE experiment was also sequenced. In total, 36 SNPs were discovered, of which three were shared among all strains and, thus, represent differences between the genome sequence of the *B. methanolicus* WT inoculum used here and the published genome sequence for *B. methanolicus* WT (Figure 5A and Tables 3, 4). Interestingly, AVA6 and AVA10 did not share additional mutations. One intragenic and one intergenic SNP were unique to AVA6. The intragenic SNP led to amino acid exchange R276I in an IS21 family transposase (BMMGA3_RS03550). The intergenic insertion was found at position 1,771,074 of the genome, i.e., 15 bp downstream of BMMGA3_RS08610 coding for deferrochelatase/peroxidase EfeB and 17 bp upstream of BMMGA3_RS08605 coding for a Fe^2+^/Pb^2+^ permease (Table 4). To gain more insight into this SNP, computational promoter and RBS analyses were performed with the prediction tools BPROM^3^ and UTR designer^4^, respectively (Supplementary Table 3). The insertion into the 32-bp-long 5′-UTR of BMMGA3_RS08605 occurred 3 bp before the predicted RBS (AGGAGT) and thus potentially interfered with translation initiation.

**TABLE 3:** Gene *loci* with SNPs in *B. methanolicus* ALE strains AVA6 and AVA10.

Locus tag	Strain	Annotation	Amino acid exchange
BMMGA3_RS03550	AVA6	IS21 family transposase	R276I
BMMGA3_RS01345	AVA10	Phosphoserine phosphatase	G132R
BMMGA3_RS03040	AVA10	Peptide MFS transporter	T132I
BMMGA3_RS03945	AVA10	3′–5′ Exoribonuclease YhaM	K109Q
BMMGA3_RS10395	AVA10	Group II intron reverse transcriptase	R219R
BMMGA3_RS13980	AVA10	FepB-type iron hydroxamate siderophore (COG0614)	*150E*^a^*
BMMGA3_RS14080	AVA10	IscR family transcriptional regulator (COG1959) of the Rrf2 superfamily	H116N
BMMGA3_RS14555	AVA10	DNA-binding response regulator	L182R
BMMGA3_RS15540	AVA10	Stage II sporulation protein R	S15S
BMMGA3_RS15615	AVA10	methylmalonyl-CoA mutase	D80Y

**^*a*^*Reverted in-frame stop codon. Intergenic SNPs, SNPs in rRNA genes or shared with the WT inoculum, are not listed.*

**TABLE 4:** Intergenic SNPs in *B. methanolicus* ALE strains AVA6 and AVA10.

Location	Strain	Description	Nucleotide exchange
1,771,074	AVA6	between BMMGA3_RS08605 (Fe^2+^/Pb^2+^ permease) and BMMGA3_RS08610 (deferrochelatase/peroxidase EfeB)	Insertion (A)
290,276	AVA10	between BMMGA3_RS01610 (transcription antiterminator) and BMMGA3_RS01615 (PTS glucose transporter subunit)	Substitution (A to G)
1,264,217	AVA10	between BMMGA3_RS06260 (BMP family ABC transporter substrate-binding protein) and BMMGA3_RS06265 (ABC transporter ATP-binding protein)	Substitution (C to G)
3,002,923	AVA10	between BMMGA3_RS14650 (undecaprenyl/decaprenyl-phosphate alpha-*N*-acetylglucosaminyl L-phosphate transferase) and BMMGA3_RS14655 (accessory Sec system translocase SecA2)	Substitution (T to G)

Curiously, AVA10 contained an SNP in gene *locus* BMMGA3_RS03040 coding for a peptide major facilitator superfamily (MFS) transporter. The members of this family are membrane proteins, and the amino acid exchange T132I caused a breakage of a buried hydrogen bond in one of the transmembrane helices (Figure 5B). Three SNPs occurred in genes, which were differentially expressed in the RNA-Seq experiment, of which two appeared in strain AVA10 (*loci* BMMGA3_RS13980 and BMMGA3_RS14080). The former SNP caused the reversion of a stop codon to a glutamate residue at position 150 (Figure 5C) identical to the related strain *B. methanolicus* PB1 (NCIMB13113). Therefore, we tentatively named the mutant protein FepB^*150E^ (Supplementary Table 2). The amino acid exchange H116N in the IscR family transcriptional regulator coded by BMMGA3_RS14080 occurred in an α-helix (Figure 5D). The mutant protein was appropriately named IscR^H116N^ (Supplementary Table 2). Additionally, three intergenic mutations occurred in strain AVA10 at positions 290,276, 1,264,217, and 3,002,923 in the genome (Table 4). As of writing this manuscript, the lack of functional tools for targeted genome editing of *B. methanolicus* impeded a further analysis of these intergenic mutations as well as the one found in AVA6. Thus, we focused on the mutations in the three genes BMMGA3_RS03040, BMMGA3_RS13980, and BMMGA3_RS14080 found in AVA10.

**FIGURE 6:**
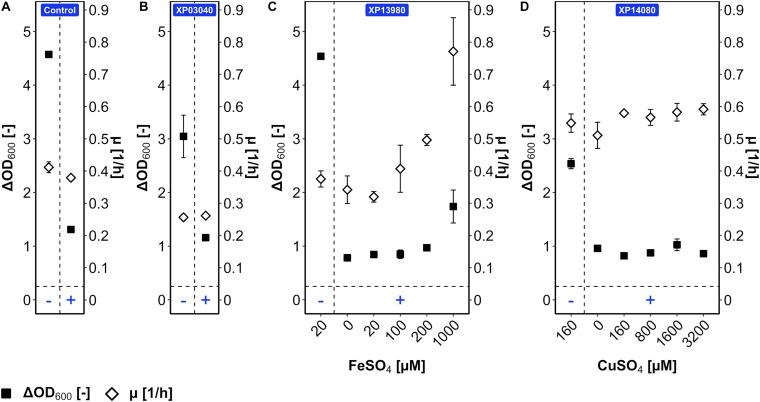
Tolerance of *B. methanolicus* WT (pBV2xp) **(A)**, XP03040 **(B)**, XP13980 **(C)**, and XP14080 **(D)** for 5AVA with cofactor titration. *B. methanolicus* cells were cultivated in MVcMY in the presence (+) and absence (−) of 50 mM 5AVA. The growth rates (empty diamonds) and ΔOD_600_ (full squares) were determined. The cofactors FeSO_4_ (0–1,000 μM) and CuSO_4_ (0–3,200 μM) were titrated for strains XP13980 **(C)** and XP14080 **(D)**, respectively, according to the function of proteins coded by their overexpressed genes and ligand prediction with COACH-D. Values and error bars represent means and standard deviations of triplicate cultivations.

The mutant versions of genes BMMGA3_RS03040, BMMGA3_RS13980, and BMMGA3_RS14080 comprising the SNPs T132I, ^∗^150E, and H116N, respectively, were cloned into plasmid pBV2xp, and *B. methanolicus* WT was transformed with the constructed vectors, resulting in the strains XP03040, XP13980, and XP14080, respectively. WT (pBV2xp) was used as an empty vector control. The overexpression analysis was conducted in the presence and absence of 50 mM 5AVA (Figure 6). The COACH-D output (Supplementary Data) hinted at Fe^2+^ and Cu^2+^ as possible cofactors for the iron siderophore-binding protein and for the Rrf2 transcriptional regulator; therefore, FeSO_4_ and CuSO_4_, which already were ingredients of the MVcM formula, were titrated, respectively. In order to rule out a growth effect solely based on the supplementation of the cofactors, *B. methanolicus* strains WT and AVA10 were cultivated and supplemented with elevated levels of the proposed ligands (Supplementary Figure 2). No significant alteration of ΔOD_600_ and growth rate was observed with the addition of 1,000 μM FeSO_4_ (Supplementary Figure 2) and 3,200 μM CuSO_4_ (Supplementary Figure 2), respectively. Cultivation of XP03040 revealed a similar growth pattern when compared to the control (Figure 6A). Similarly, in the presence of 5AVA, strain XP14080 did not grow to higher ΔOD_600_ compared with the control regardless of the CuSO_4_ concentration (Figure 6D). Thus, since their overexpression did not improve 5AVA tolerance, the respective SNPs present in AVA10 (leading to amino acid exchanges H116N for Rrf2 family transcriptional regulator and T132I for peptide MFS transporter) are not primarily responsible for improved 5AVA tolerance of the ALE strain AVA10.

By contrast, while strain XP13980 grew like the empty vector control in the absence and presence of 50 mM 5AVA, increasing the iron concentration in the presence of 5AVA resulted in a 126% higher growth rate (0.77 ± 0.10 vs. 0.34 ± 0.04 h^–1^) and 123% higher ΔOD_600_ (1.7 ± 0.3 vs. 0.8 ± 0.0) (Figure 6C). The maximal ΔOD_600_ of strain XP13980 (1.7 ± 0.3) was still lower than that of AVA10 (2.9 ± 0.1) (Figure 1B). Notably, the growth rate of XP13980 (0.77 ± 0.10 h^–1^) surpassed that of ALE strain AVA10 (0.62 ± 0.00 h^–1^) (Figure 1B). Thus, the increased tolerance of AVA10 to 5AVA is possibly due to the SNP in BMMGA3_RS13980 that reverted a stop codon at position 150, leading to the full-length iron siderophore-binding protein. As the native BMMGA3_RS13980 was not characterized in the same experiment, higher tolerance due to overexpression of the non-mutant version or a combinatorial effect is possible. Commensurate with its function in iron acquisition, increased Fe^2+^ supplementation improved growth of strains AVA10 and XP13980 in the presence of 5AVA, but not of WT that lacked the full-length iron siderophore-binding protein.

## Discussion

Among the tested short-chain (C4–C6) amino acids, diamines, and dicarboxylic acids, extracellularly added 5AVA already impaired growth of *B. methanolicus* at 10 mM. In the presence of 5AVA, the general stress response was triggered, and RNA levels of iron transport genes (BMMGA3_RS13980 and BMMGA3_RS13970) were increased, while the gene coding for an Rrf2 family transcriptional regulator (BMMGA3_RS14080) showed reduced levels. Notably, mutants with increased 5AVA tolerance selected by ALE carried SNPs in two of these genes (BMMGA3_RS14080 and BMMGA3_RS13980). Moreover, overexpression of the mutant version of BMMGA3_RS13980 in combination with Fe^2+^ supplementation improved 5AVA tolerance of *B. methanolicus* almost to the level of the ALE mutant.

5-Aminovalerate tolerance has been studied in *C. glutamicum*, which, however, was hardly affected by 5AVA with a half-maximal inhibitory concentration (IC_50_) of 1.1 M (Jorge et al., 2017), i.e., about three orders of magnitude higher than the inhibition observed here for *B. methanolicus* (Figure 1A). Both bacteria share neither being able to catabolize 5AVA nor use it as a carbon or nitrogen source (Jorge et al., 2017; Figure 2). GABA, the C4 structural homolog of 5AVA, was less inhibitory on the growth of *B. methanolicus* with an IC_50_ ∼70 mM (Irla et al., 2017). However, *C. glutamicum* showed a still higher IC_50_ of ∼1.1 M (Jorge et al., 2016). Similarly, high tolerance against 6ACA, the C6 structural homolog of 5AVA, has been reported for *B. subtilis* and *E. coli* (Turk et al., 2016). Strain AVA10 that was selected here for higher 5AVA tolerance actually was less tolerant to both GABA and 6ACA (Figure 1). Thus, the stress due to 5AVA addition and the tolerance selected by ALE was specific to 5AVA and differed from effects due to addition of the ω-amino acids GABA and 6ACA.

5-Aminovalerate-specific gene expression changes were due to the general stress response, but additional effects were observed as well. Reduced RNA levels of flagellation, pyrimidine biosynthesis, and ribosomal function genes in tandem with increased RNA levels of sporulation targets commonly characterize the general stress response (Eymann et al., 2002; Han et al., 2017). In the related *B. subtilis*, the general stress response is triggered by activation of the sigma factor σ^B^ and is distinct from the stringent response mediated via (p)ppGpp and amino acid starvation (Eymann et al., 2002). It can be hypothesized that the general stress response is regulated in a similar manner in *B. methanolicus* as in *B. subtilis*, since sporulation and biofilm formation are controlled by Spo0A in both bacilli (Piggot and Hilbert, 2004; Schultenkämper et al., 2019). Induced expression of histidine biosynthesis genes observed in the presence of 5AVA for *B. methanolicus* (Figures 2, 3B), but not *C. glutamicum* (Jorge et al., 2017), prompted us to speculate if histidine and proline may be converted in a similar manner as in the Stickland reaction that is typically observed in proteolytic clostridia (Barker et al., 1987). The uptake and catabolism of toxic compounds is a regular vent for microorganisms in toxic environments, e.g., chemical herbicides and propionate (Huang et al., 2017; Dolan et al., 2018). However, 5AVA was catabolized neither by *B. methanolicus* WT nor by ALE strain AVA10, and its concentration in the culture medium remained unchanged during cultivation (Figure 3B). Also, no 5AVA was measured in metabolic studies of *B. methanolicus* (Carnicer et al., 2016; Delépine et al., 2020). It is unclear if 5AVA lacks a system for import of 5AVA into the *B. methanolicus* cell or whether it lacks genes for 5AVA catabolism. Inspection of the genome sequence did not reveal homologs of the *gabTDP* operon for import and degradation of 5AVA, which were found in *C. glutamicum*, *E. coli*, and *Pseudomonas putida* (Espinosa-Urgel and Ramos, 2001; Jorge et al., 2017; Knorr et al., 2018). Regulatory effects due to 5AVA that do not affect genes for uptake and catabolism may exist. With respect to amino acid transport, this is known for the lysine, arginine, and citrulline export system LysE of *C. glutamicum* (Vrljic et al., 1996; Lubitz et al., 2016), which does not accept histidine as a substrate. However, histidine is a coactivator of the transcriptional activator protein LysG that activates transcription of the *lysE* gene (Bellmann et al., 2001).

5-Aminovalerate tolerance was shown to be associated with iron acquisition, and it could be increased by overexpression of an allele of BMMGA3_RS13980 that was isolated by ALE and occurred in strain AVA10. Independently, strain AVA6 possessed an intergenic mutation between genes coding for a putative deferrochelatase and a putative Fe^2+^/Pb^2+^ permease (BMMGA3_RS08610 and BMMGA3_RS08605; Figure 5A). In *B. methanolicus* WT, RNA levels of BMMGA3_RS13980 and a second gene involved in iron acquisition, BMMGA3_RS13970, were increased in response to extracellular 5AVA (Supplementary Tables 1, 3). Iron acquisition plays an important role in the physiology of microorganisms and plants, especially when competing with other organisms and involving iron-chelating agents and biosynthetic chelators called siderophores (Crowley et al., 1991). For example, *C. glutamicum*, which neither synthesizes nor secretes a siderophore, grows faster in the presence of exogenous iron chelators (Liebl et al., 1989), although they are not strictly required (Graf et al., 2019). In *C. glutamicum*, extracellularly added indole was shown to inhibit growth, to increase expression of iron acquisition genes, and to chelate iron, and a mutant of the iron homeostasis regulator DtxR isolated by ALE improved indole tolerance (Walter et al., 2020). The genome of *B. methanolicus* does not code for a DtxR homolog according to BLASTx analysis; however, the study presented here also identifies involvement of a regulatory gene, BMMGA3_RS14080, coding for an Rrf2 family transcriptional regulator. Currently, it is unknown if iron or 5AVA affects this regulator. Some Rrf2 family transcriptional regulators bind to small-molecule ligands as effectors (Shepard et al., 2011). For example, Rrf2 family transcriptional regulators Rrf2 from *Desulfovibrio vulgaris*, IscR from *E. coli*, and RirA from *Rhizobiales* and other α-proteobacteria regulate biogenesis of Fe–S clusters and Fe–S cluster-containing proteins and in response to the demand for 2Fe–2S clusters (Keon et al., 1997; Giel et al., 2013).

The mechanism by which 5AVA alters iron availability or acquisition remains to be elucidated. Curiously, 5AVA inhibits the *N*^5^-hydroxylation of ornithine by PvdA from *Pseudomonas aeruginosa* with a K_ic_s of 2.9 ± 0.3 mM (Ge and Seah, 2006). The pathogen *P. aeruginosa* acquires iron from its host by synthesizing the hydroxamate siderophore pyoverdine, which involves the flavin-dependent monooxygenase PvdA (Ge and Seah, 2006; Peek et al., 2012). In *B. subtilis*, the hydroxamates schizokinen, ferrichrome, and ferrioxamine E were detected (Byers, 1974). *B. methanolicus* possesses genes BMMGA3_RS15920, BMMGA3_RS15925, BMMGA3_RS15930, and BMMGA3_RS15935 coding for ferrichrome ABC transporter ferrichrome-binding protein, Fe^3+^-hydroxamate import system permease proteins FhuB and FhuG, and Fe^3+^-hydroxamate import ATP-binding protein FhuC, respectively. BLAST analysis does not reveal *pvdA* homologs in *B. methanolicus*; however, *B. subtilis* possesses a lysine *N*^6^-hydroxylase/L-ornithine *N*^5^-oxygenase family protein and a SidA/IucD/PvdA family monooxygenase (Acc.: WP_025811671.1 and WP_072588947.1) as a first step for hydroxamate synthesis. Characterization of siderophore production and more specifically hydroxamate production in *B. methanolicus* could provide further insight into the use of the hydroxamate siderophore ferrichrome and possibly the inhibition of ferrichrome biosynthesis by 5AVA.

Taken together, ALE, in particular in combination with transcriptomics, is suitable to select mutants with increased tolerance toward substrates, e.g., methanol (Tuyishime et al., 2018; Hennig et al., 2020; Wang et al., 2020), and products, e.g., indole (Walter et al., 2020). Genome sequencing of ALE strains is straightforward, identifying mutations in genes that may be relevant for the selected phenotype. For microorganisms amenable to gene deletion or gene replacement, reverse engineering of the identified mutations into the parental strain to identify causal mutations among the candidates identified by genome sequencing is the strategy of choice (Hennig et al., 2020; Walter et al., 2020). Due to the lack of genome editing tools for *B. methanolicus*, we opted to analyze strains overexpressing the mutant genes identified by genome sequencing (Figures 6A–D) in a similar manner as used, e.g., for validating improved cadmium tolerance observed in ALE of a cyanobacterium (Xu et al., 2018). Concerning the analysis of the AVA6 intergenic mutation (Supplementary Table 3), it is possible to further investigate this SNP without the use of genome editing. This includes the cloning of native and mutant versions of 5′-/3′-UTRs or promoter regions into plasmids for transcriptional and translational fusions with reporter genes to evaluate their expression strength. Furthermore, binding motifs interacting with known or unknown regulators may be found by using bandshift assays or ChIP-seq, respectively. However, the recent development of CRISPRi-mediated gene repression in *B. methanolicus* (Schultenkämper et al., 2019) foreshadows the future development of CRISPR-based genome editing, which will ease deciphering causal mutations in a pool of mutations identified by genome sequencing of ALE strains.

## Data Availability Statement

The datasets presented in this study can be found in online repositories. The names of the repository/repositories and accession number(s) can be found in the article/Supplementary Material.

## Author Contributions

CH and VW conceptualized this work and reviewed and edited the manuscript. CH constructed strains and drafted the manuscript. CH, LB, and TB performed the experiments. CH, LB, TB, DW, and VW analyzed the data. VW finalized the manuscript. All authors agreed to the final version of the manuscript.

## Conflict of Interest

The authors declare that the research was conducted in the absence of any commercial or financial relationships that could be construed as a potential conflict of interest.

## Footnotes

1 https://www.european-bioplastics.org/market/ (accessed on 03.12.2020)

2 https://www.methanex.com/our-business/pricing (accessed on 03.12.2020)

3 http://www.softberry.com/berry.phtml?topic=bprom&group=programs&subgroup=gfindb

4 https://sbi.postech.ac.kr/utr_designer/reverse/
