# Supplementary material for: Genomic and Transcriptomic Investigation of the Physiological Response of the Methylotroph Bacillus methanolicus to 5-Aminovalerate
Source: Front Microbiol. 2021 Apr 30;12:664598. doi: 10.3389/fmicb.2021.664598 (PMC8119775; doi:10.3389/fmicb.2021.664598)
Supplement: Supplementary file 1 [file Data_Sheet_1.PDF]

Supplementary Material

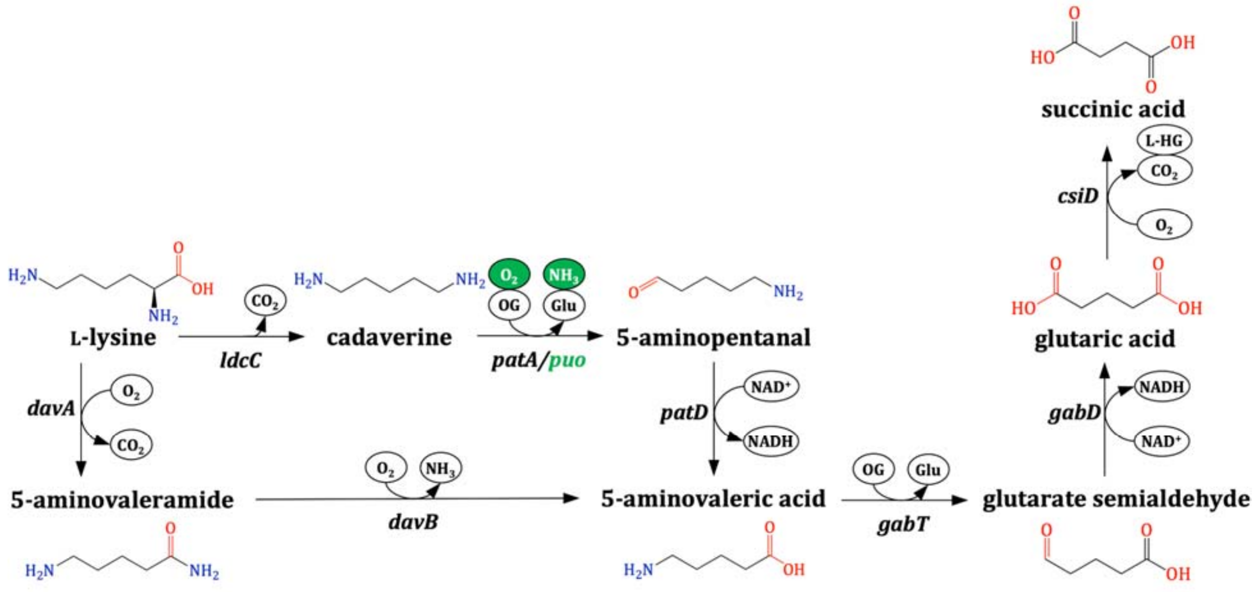

Figure S1. The involvement of 5-aminovaleric acid (5AVA) in L-lysine degradation/conversion. Genes and their gene products: *csiD*, glutarate hydroxylase; davA, L-lysine monoxygenase; *davB*, 5-aminovaleramidase; gabT, γ-aminobutyrate (GABA)/5AVA amino transferase; *gabD*, succinate/glutarate-semialdehyde dehydrogenase; *ldcC*, L-lysine decarboxylase; *patA*, putrescine transaminase; *patD*, γ-aminobutyraldehyde dehydrogenase; *puo*, putrescine oxidase; metabolites: L-HG, L-2-hydroxyglutaric acid; OG, 2-oxoglutarate; Glu, L-glutamatic acid.

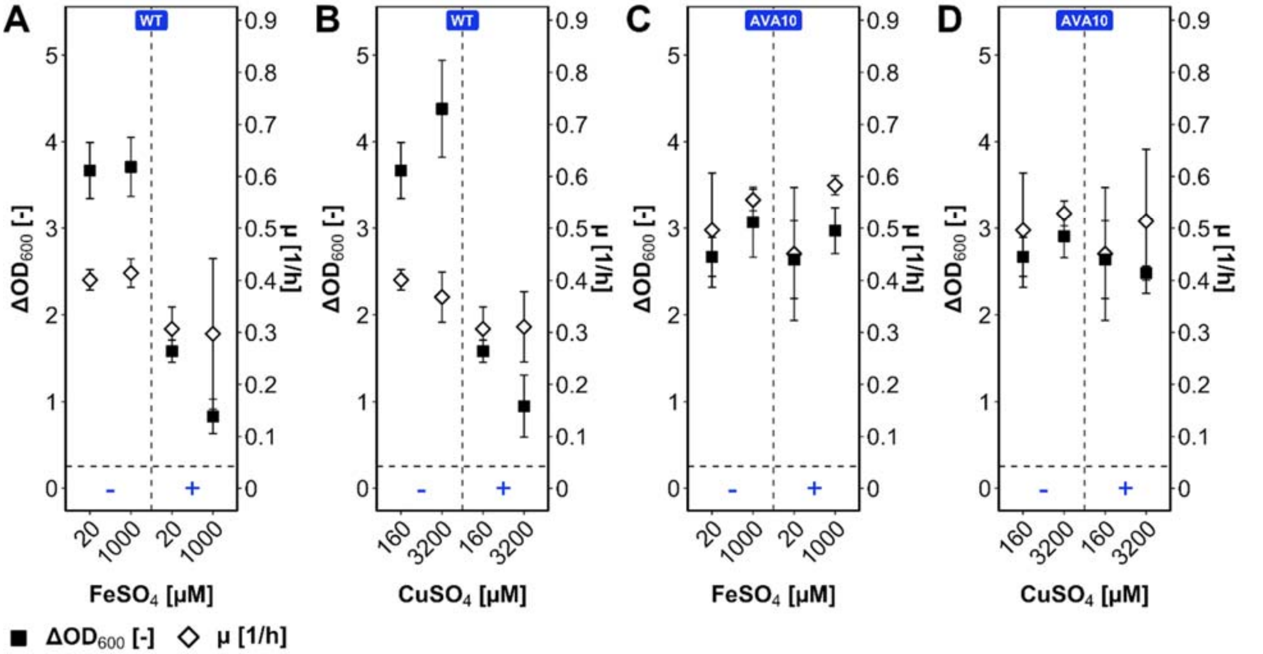

Figure S2. Tolerance of *B. methanolicus* WT (**A, B**) and AVA10 (**C, D**) for 5AVA with cofactor titration. *B. methanolicus* cells were cultivated in MVcMY in the presence (+) and absence (-) of 50 mM 5AVA, respectively. The growth rates (empty diamonds) and ΔOD_{600} (full squares) were determined. The cofactors $FeSO_4$ (20 µM, 1000 µM; **A, C**) and $CuSO_4$ (160 µM, 3200 µM; **B, D**) were titrated for both strains. Values and error bars represent mean and standard deviation of triplicate cultivations.

Table S1. Differentially expressed genes of *B. methanolicus* WT with a M-value > |1| cultivated with/without 50 mM 5AVA.

Locus tag	Annotation	Log2 fold change of RNA level[a]
BMMGA3_RS00230	AbrB/MazE/SpoVT family DNA-binding domain-containing protein	-1.57
BMMGA3_RS00250	ribonuclease M5	1.53
BMMGA3_RS00255	16S rRNA (adenine(1518)-N(6)/adenine(1519)-N(6))- dimethyltransferase RsmA	1.32
BMMGA3_RS00260	sporulation peptidase YabG	1.52
BMMGA3_RS00370	stage II sporulation protein E	1.95
BMMGA3_RS00375	hypothetical protein	2.24
BMMGA3_RS00380	serine/threonine protein kinase	2.38
BMMGA3_RS00545	CtsR family transcriptional regulator	1.29
BMMGA3_RS00550	UvrB/UvrC motif-containing protein BLAST	1.27
BMMGA3_RS00555	protein arginine kinase	1.27
BMMGA3_RS01500	pilus biosynthesis protein CpaE	1.33
BMMGA3_RS01505	CpaF family protein	1.36
BMMGA3_RS01510	membrane protein	1.49

BMMGA3_RS01515	pilus assembly protein TadB	1.52
BMMGA3_RS01520	hypothetical protein	1.44
BMMGA3_RS01525	VWA domain-containing protein	1.40
BMMGA3_RS01530	hypothetical protein	1.70
BMMGA3_RS01535	hypothetical protein	1.23
BMMGA3_RS01540	hypothetical protein	1.19
BMMGA3_RS01545	hypothetical protein	1.24
BMMGA3_RS01550	DUF4234 domain-containing protein BLAST	1.41
BMMGA3_RS01560	hypothetical protein	1.36
BMMGA3_RS01565	VWA domain-containing protein BLAST	1.35
BMMGA3_RS02005	bacteriocin	1.20
BMMGA3_RS02820	sugar ABC transporter permease	1.35
BMMGA3_RS02825	carbohydrate ABC transporter permease	1.27
BMMGA3_RS02830	family 65 glycosyl hydrolase	1.47
BMMGA3_RS03270	zinc ABC transporter substrate-binding protein	-1.24
BMMGA3_RS03365	hypothetical protein	1.24
BMMGA3_RS04320	GTP 3',8-cyclase MoaA	1.19
BMMGA3_RS04325	molybdopterin molybdenumtransferase MoeA	1.37
BMMGA3_RS04330	molybdopterin-guanine dinucleotide biosynthesis protein B	1.30
BMMGA3_RS04345	acetylglutamate kinase	1.18
BMMGA3_RS04650	EAL domain-containing protein	-2.59
BMMGA3_RS04655	PilZ domain-containing protein	-2.33
BMMGA3_RS04980	phosphocarrier protein HPr	-1.10
BMMGA3_RS05175	nitronate monooxygenase	1.30
BMMGA3_RS05265	hypothetical protein	-1.21
BMMGA3_RS05415	acetyl-CoA carboxylase biotin carboxylase subunit	1.11
BMMGA3_RS11270	DUF4912 domain-containing protein	1.05
BMMGA3_RS05420	acetyl-CoA carboxylase biotin carboxyl carrier protein subunit	1.21
BMMGA3_RS05535	sigma-E processing peptidase SpoIIGA	1.60
BMMGA3_RS05540	RNA polymerase sporulation sigma factor SigE	1.73
BMMGA3_RS05545	RNA polymerase sporulation sigma factor SigG	1.62
BMMGA3_RS05550	YlmC/YmxH family sporulation protein	2.06
BMMGA3_RS05605	bifunctional pyr operon transcriptional regulator/uracil phosphoribosyltransferase PyrR	-1.16
BMMGA3_RS05610	uracil transporter	-2.48
BMMGA3_RS05615	aspartate carbamoyltransferase catalytic subunit	-2.77
BMMGA3_RS05620	dihydroorotase	-3.04
BMMGA3_RS05625	carbamoyl-phosphate synthase small subunit	-3.03
BMMGA3_RS05630	carbamoyl-phosphate synthase large subunit	-3.16
BMMGA3_RS10115	DUF1798 domain-containing protein	1.15
BMMGA3_RS05635	dihydroorotate dehydrogenase electron transfer subunit	-3.11
BMMGA3_RS05640	dihydroorotate dehydrogenase	-3.59
BMMGA3_RS05645	orotidine-5'-phosphate decarboxylase	-3.29
BMMGA3_RS05650	orotate phosphoribosyltransferase	-3.23
BMMGA3_RS05925	flagellar basal body rod protein FlgB	-1.41
BMMGA3_RS13960	DUF2573 domain-containing protein	1.18
BMMGA3_RS05930	flagellar basal body rod protein FlgC	-1.26
BMMGA3_RS06340	2-oxoacid:acceptor oxidoreductase subunit alpha	1.08
BMMGA3_RS06345	2-oxoacid ferredoxin oxidoreductase subunit beta	1.16
BMMGA3_RS06535	sporulation inhibitor of replication protein SirA	1.22
BMMGA3_RS07325	transcription antiterminator BglG	2.01
BMMGA3_RS07330	PTS sugar transporter subunit IIA	2.28
BMMGA3_RS07335	PTS galactitol transporter subunit IIB	1.76
BMMGA3_RS07340	PTS galactitol transporter subunit IIC	2.02
BMMGA3_RS07345	sorbitol dehydrogenase	2.28
BMMGA3_RS07350	hypothetical protein	2.23
BMMGA3_RS07355	galactitol-1-phosphate 5-dehydrogenase	1.45
BMMGA3_RS07735	hypothetical protein	1.57

BMMGA3_RS07745	hypothetical protein	1.58
BMMGA3_RS07750	DNA-binding response regulator	1.87
BMMGA3_RS07755	sensor histidine kinase	2.01
BMMGA3_RS08045	aldehyde dehydrogenase family protein	1.18
BMMGA3_RS08160	2-oxoglutarate dehydrogenase E1 component	1.11
BMMGA3_RS08555	MerR family transcriptional regulator BLAST	1.79
BMMGA3_RS08605	FTR1 family protein BLAST	1.01
BMMGA3_RS08650	orotidine 5'-phosphate decarboxylase	1.03
BMMGA3_RS08655	nicotinate phosphoribosyltransferase	1.01
BMMGA3_RS09130	ABC transporter ATP-binding protein	1.20
BMMGA3_RS09235	tryptophan synthase subunit beta	1.22
BMMGA3_RS09240	phosphoribosylanthranilate isomerase	1.24
BMMGA3_RS09245	indole-3-glycerol phosphate synthase TrpC	1.26
BMMGA3_RS13955	DUF2071 domain-containing protein	1.30
BMMGA3_RS09250	anthranilate phosphoribosyltransferase	1.22
BMMGA3_RS09255	type 1 glutamine amidotransferase	1.44
BMMGA3_RS09260	anthranilate synthase component I	1.21
BMMGA3_RS09300	PTS fructose transporter subunit IIABC	1.41
BMMGA3_RS09305	1-phosphofructokinase	1.28
BMMGA3_RS10105	YppG family protein BLAST	1.38
BMMGA3_RS10120	hypothetical protein	1.87
BMMGA3_RS10630	RNA polymerase sporulation sigma factor SigF	1.16
BMMGA3_RS10635	anti-sigma F factor	1.32
BMMGA3_RS10640	anti-sigma F factor antagonist	1.14
BMMGA3_RS10910	sigma-54-dependent Fis family transcriptional regulator	1.07
BMMGA3_RS11260	hypothetical protein	1.75
BMMGA3_RS11265	glycosyl transferase	1.65
BMMGA3_RS11865	N-acetylmuramoyl-L-alanine amidase	1.02
BMMGA3_RS12035	50S ribosomal protein L27	-1.22
BMMGA3_RS12040	ribosomal-processing cysteine protease Prp	-1.04
BMMGA3_RS12100	hypothetical protein	1.66
BMMGA3_RS12160	hypothetical protein	-1.17
BMMGA3_RS12285	3-isopropylmalate dehydratase large subunit	1.04
BMMGA3_RS12295	2-isopropylmalate synthase	1.13
BMMGA3_RS12305	acetolactate synthase small subunit	1.01
BMMGA3_RS12375	succinate dehydrogenase flavoprotein subunit	1.03
BMMGA3_RS12540	S-adenosylmethionine decarboxylase proenzyme	-1.29
BMMGA3_RS13250	glycogen/starch/alpha-glucan phosphorylase	1.70
BMMGA3_RS13255	glycogen synthase GlgA	1.90
BMMGA3_RS13260	glucose-1-phosphate adenylyltransferase	1.91
BMMGA3_RS13265	glucose-1-phosphate adenylyltransferase	1.95
BMMGA3_RS13270	1,4-alpha-glucan branching protein GlgB	1.65
BMMGA3_RS13815	branched-chain amino acid ABC transporter permease	1.03
BMMGA3_RS13820	branched-chain amino acid ABC transporter permease	1.18
BMMGA3_RS13825	ABC transporter ATP-binding protein	1.15
BMMGA3_RS13830	ABC transporter ATP-binding protein	1.15
BMMGA3_RS13950	FMN-binding glutamate synthase family protein	1.41
BMMGA3_RS13965	ABC transporter ATP-binding protein	1.31
BMMGA3_RS13970	iron ABC transporter permease	1.15
BMMGA3_RS13980	iron siderophore-binding protein	1.56
BMMGA3_RS13985	spore germination protein	1.56
BMMGA3_RS13990	spore gernimation protein GerB	1.06
BMMGA3_RS14080	Rrf2 family transcriptional regulator	-1.04
BMMGA3_RS14230	bifunctional phosphoribosyl-AMP cyclohydrolase/phosphoribosyl-ATP diphosphatase HisIE	1.12
BMMGA3_RS14235	imidazole glycerol phosphate synthase subunit HisF	1.37

BMMGA3_RS14240	1-(5-phosphoribosyl)-5-[(5-phosphoribosylamino)methylideneamino]imidazole-4- carboxamide isomerase	1.30
BMMGA3_RS14245	imidazole glycerol phosphate synthase subunit HisH	1.32
BMMGA3_RS14250	imidazoleglycerol-phosphate dehydratase HisB	1.32
BMMGA3_RS14255	histidinol dehydrogenase	1.50
BMMGA3_RS14260	ATP phosphoribosyltransferase	1.48
BMMGA3_RS14265	ATP phosphoribosyltransferase regulatory subunit	1.28
BMMGA3_RS14530	membrane protein	-1.21
BMMGA3_RS14665	endopeptidase LytE	-1.28
BMMGA3_RS14760	methyl-accepting chemotaxis protein	-1.22
BMMGA3_RS14770	hypothetical protein	-1.32
BMMGA3_RS15115	hypothetical protein	-1.70
BMMGA3_RS15250	NlpC/P60 family protein	-1.35
BMMGA3_RS15420	NAD(P)H-quinone oxidoreductase subunit H	1.17
BMMGA3_RS15435	NADH-quinone oxidoreductase subunit A	1.07
BMMGA3_RS15470	F0F1 ATP synthase subunit C	-1.07
BMMGA3_RS16675	(2Fe-2S)-binding protein BLAST	1.01
BMMGA3_RS17295	DNA-binding anti-repressor SinI	1.06

[a] Cut-off values set to a change in expression level higher than 30; $P \leq 0.01$, determined by DESeq2 according to the Wald test (Love et al., 2014).

Table S2. Gene and protein links provided for key genes in *B. methanolicus* AVA10 which gained mutations through ALE.

Locus tag	(Mutant) Protein name	Gene link	Protein link-
BMMGA3_RS03040	-	https://www.ncbi.nlm.nih.gov/nuccore/NZ_CP007739.1?from=610932&to=612425	https://www.ncbi.nlm.nih.gov/protein/WP_003347912.1
BMMGA3_RS13980	FepB$^{(*150E)}$	https://www.ncbi.nlm.nih.gov/nuccore/NZ_CP007739.1?from=2858093&to=2859088	-
BMMGA3_RS14080	IscR$^{(H116N)}$	https://www.ncbi.nlm.nih.gov/nuccore/NZ_CP007739.1?from=2879155&to=2879565	https://www.ncbi.nlm.nih.gov/protein/WP_003349446.1/

Table S3. 5'-UTR of BMMGA3_RS08605 in *B. methanolicus* with the insertion at position 1771074. Promoter and RBS prediction were made with BPROM and UTR designer, respectively.

5'-Sequence-3' (-35/-10 Box, RBS, Gene, Insertion)
GGGATATATTGGAGAAACGCTATTTTCATAAGCCGTTTTTTTTTTTTTCATAGGAGTTGATAATAATGTAT AAAAAAATTTGCCTTCTTGCCTTCGTGTT
